# *Hox* genes reveal genomic DNA variation in tetraploid hybrids derived from *Carassius auratus* red var. (female) × *Megalobrama amblycephala* (male)

**DOI:** 10.1186/s12863-017-0550-2

**Published:** 2017-10-11

**Authors:** Y. D. Wang, Q. B. Qin, R. Yang, W. Z. Sun, Q. W. Liu, Y. Y. Huo, X. Huang, M. Tao, C. Zhang, T. Li, S. J. Liu

**Affiliations:** 1 State Key Laboratory of Developmental Biology of Freshwater Fish, Hunan Normal University, Changsha, 410081 Hunan People’s Republic of China; 2 College of Life Sciences, Hunan Normal University, Changsha, 410081 Hunan People’s Republic of China

**Keywords:** Allotetraploid, *Hox gene*, polyploidization, pseudogenization

## Abstract

**Background:**

Allotetraploid F_1_ hybrids (4nF_1_) (AABB, 4n = 148) were generated from the distant hybridization of *Carassius auratus* red var. (RCC) (AA, 2n = 100) (♀) × *Megalobrama amblycephala* (BSB) (BB, 2n = 48) (♂). It has been reported that *Hox* gene clusters are highly conserved among plants and vertebrates. In this study, we investigated the genomic organization of *Hox* gene clusters in the allotetraploid F_1_ hybrids and their parents to investigate the polyploidization process.

**Results:**

There were three copies of *Hox* genes in the 4nF_1_ hybrids, two copies in RCC and one copy in BSB. In addition, obvious variation and pseudogenization were observed in some *Hox* genes from 4nF_1._

**Conclusion:**

Our results reveal the influence of polyploidization on the organization and evolution of *Hox* gene clusters in fish and also clarify some aspects of vertebrate genome evolution.

**Electronic supplementary material:**

The online version of this article (10.1186/s12863-017-0550-2) contains supplementary material, which is available to authorized users.

## Background

Polyploidization is a widespread mechanism for speciation in eukaryotes, especially plants and vertebrates [1–5]. Polyploids with duplicated genomes may originate from a single species (autopolyploidy) or from different species through interspecific hybridization (allopolyploidy) [6]. Allopolyploids are prevalent in nature, suggesting there is an evolutionary advantage to obtaining multiple sets of genetic material for adaptation and development [7]. However, the molecular mechanisms underlying the processes and consequences of allopolyploidy remain unclear [8]. Polyploidy is relatively rare in animals compared with plants, and the influence of polyploidization on intragenomic variation in polyploid animals is poorly understood. In our earlier study, we successfully obtained fertile tetraploid hybrids from *Carassius auratus* red var. (RCC)(♀) × *Megalobrama amblycephala* (BSB)(♂) [9, 10]. RCC has 100 chromosomes and belongs to the Cyprinidae subfamily, while BSB has 48 chromosomes and belongs to the Cultrinae subfamily [11]. These new polyploid hybrids represent unique specimens for studying genomic changes in F_1_ hybrids and could significantly contribute to our understanding of evolution.

*Hox* genes, a set of important developmental regulatory genes, are highly conserved and typically organized cluster [12]. In vertebrates, *Hox* genes encode two exons, and the highly conserved homeodomain (60 aa) is encoded by the second exon [13]. Recent research has shown that gene duplication, sequence variation, and selective pressure played crucial roles in the origin and evolution of *Hox* genes [14]. The earliest indications of genome duplication came from the comparative analysis of *Hox* genes and clusters from different chordate lineages [15–18].

In general, polyploidization plays an important role in fish evolution [19]. The purpose of this research was to study the effects of allopolyploidization on *Hox* gene organization and evolution. In this article, three distinct *Hox* duplicates were observed in the 4nF_1_ genome, compared with two copies in RCC and one copy in BSB. Our data reveal the genetic variation and evolutionary characteristics of the *Hox* gene family in 4nF_1_ and provide new insights into their evolutionary patterns.

## Results

### Sequence information for RCC, BSB and 4nF_1_ clones

Using 11 pairs of degenerate primers (Additional file 1: Table S1), we obtained partial sequence information for eight putative *Hox* genes from RCC, four putative *Hox* genes from BSB, and 32 putative *Hox* genes from the 4nF_1_. All these fragments were between 700 and 1500 bp long and included the exon1-intron-exon2 region (Table 1). To avoid biased amplification of only one *Hox* gene copy, we selected 20 clones of each gene from 4nF_1_, 20 clones of each gene from RCC and 80 clones from BSB (20 clones for each *Hox* gene PCR fragment). All fragments from RCC, BSB and the 4nF_1_ were confirmed to be *Hox* gene sequences, and each included the homeobox. All *Hox* sequences have been submitted to GenBank; their accession numbers are listed in Table 1.

**Table 1:** PCR amplification bands in RCC, BSB and 4nF_1_

Species	Locus	Size (bp)	Exon1 (bp)	Intron (bp)	Exon 2 (bp)	GenBank accession no.
RCC	*HoxA4ai*	1181	89-500	501-974	975-1181	JX282274
	*HoxA4aii*	1184	89-500	501-977	978-1184	JX282275
	*HoxA9ai*	867	1-381	382-670	671-867	JX282276
	*HoxA9aii*	819	1-381	382-622	623-819	JX282277
	*HoxA2bi*	1486	1-314	315-901	902-1486	JX282278
	*HoxA2bii*	1448	1-314	315-863	864-1448	JX282279
	*HoxD4ai*	960	1-315	316-735	736-960	JX282280
	*HoxD4aii*	952	1-315	316-719	728-952	JX282281
BSB	*HoxA4a*	1188	89-500	501-981	982-1188	JX282282
	*HoxA9a*	879	1-381	382-682	683-879	JX282283
	*HoxA2b*	1479	1-311	312-894	895-1479	JX282284
	*HoxD4a*	911	1-306	307-686	687-911	JX282285
4nF_1_	*HoxA4ai*	1183	89-500	501-976	977-1183	JQ901468
	*HoxA4aii*	1169	89-500	501-962	963-1169	JX282286
	*HoxA4aiii*	1177	89-500	501-970	971-1177	JX282287
	*HoxA9ai*	867	1-381	382-670	671-867	JX282288
	*HoxA9aii*	817	1-381	382-620	621-817	JX282289
	*HoxA9aiii*	863	1-381	382-666	667-863	JX282290
	*HoxA2bi*	1486	1-314	315-901	902-1486	JX282291
	*HoxA2bii*	1448	1-314	315-863	864-1448	JX282292
	*HoxA2biii*	1475	1-314	315-890	891-1475	JX282293
	*HoxA11bi*	1251	3-590	591-1153	1154-1251	JX282294
	*HoxA11bii*	1411	3-590	591-1313	1314-1411	JX282295
	*HoxA11biii*	1437	3-590	591-1339	1340-1437	JX282296
	*HoxB1bi*	733	1-477	478-567	568-733	JX282297
	*HoxB1bii*	734	1-477	478-568	569-734	JX282298
	*HoxB1biii*	731	1-477	478-565	566-731	JX282299
	*HoxB5bi*	1196	1-561	562-990	991-1196	JX282300
	*HoxB5bii*	1195	1-561	562-989	990-1196	JX282301
	*HoxB5biii*	1190	1-561	562-984	985-1190	JX282302
	*HoxB6bi*	807	1-169	170-667	668-807	JX282303
	*HoxB6bii*	819	1-169	170-679	680-819	JX282304
	*HoxB6biii*	812	1-169	170-672	673-819	JX282304
	*HoxC4ai*	1176	1-410	411-935	936-1176	JX282306
	*HoxC4aii*	1173	1-410	411-932	933-1173	JX282307
	*HoxC4aiii*	1169	1-410	411-928	929-1169	JX282308
	*HoxC4a-1*	1179	1-410	411-938	939-1179	JX282309
	*HoxD4ai*	960	1-315	316-735	736-960	JX282310
	*HoxD4aii*	952	1-315	316-719	728-952	JX282311
	*HoxD4aiii* ^Ψ^	942	-	-	-	JX282312
	*HoxD9a* ^Ψ^	897	-	-	-	JX282313
	*HoxD10a* ^Ψ^	1481	-	-	-	JX282314
	*HoxD10aii*	1554	1-589	590-1324	1325-1554	JX282315
	*HoxD10aiii*	1495	1-592	593-1265	1266-1495	JX282316

Ψ denotes a pseudogene

### Molecular organization of the *Hox* gene sequence

**Fig. 1:**
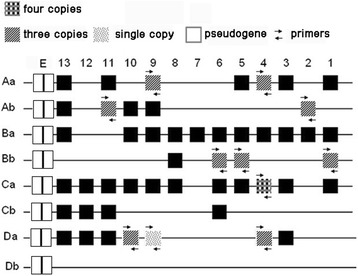
*Hox* cluster architecture in 4nF_1_ compared with zebrafish. We identified a total of 32 *Hox* genes. Nine *Hox* genes were present in three copies, one *Hox* gene was present in four copies, and one was present as a single copy in 4nF_1_. Copies of the *HoxD9a*, *HoxD4a*, and *HoxD10a* genes were pseudogenes. Black boxes represent *Hox* genes from *Danio rerio*, and “E” refers to EVX (even-skipped related gene). Aa, Ab, Ba, Bb, Ca, Cb, Da and Db refer to classes of genes

**Fig. 2:**
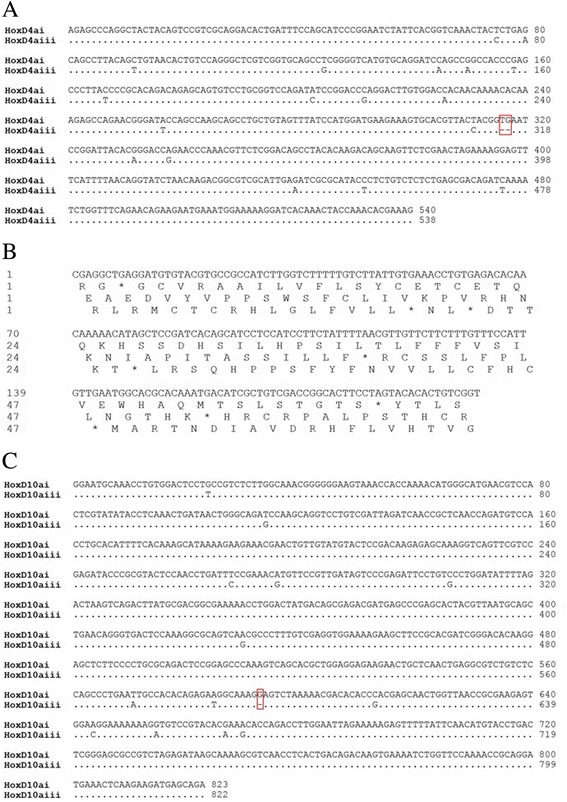
The pseudogenes *HoxD9a*, *HoxD4aiii*, and *HoxD10ai* in 4nF_1_. **a** Two deletions occurred in codons 316 and 317 in the coding region of *HoxD4aiiiΨ*. **b** *HoxD9aΨ* became a pseudogene because a stop codon prematurely terminated expression of the full-length functional product. **c** One insertion occurred in codon 593 in the coding region of *HoxD10aiΨ*

We comparatively analysed the inferred amino acid sequences of the *Hox* genes in 4nF_1_ with those in zebrafish, fugu, medaka, and BSB (Additional file 1: Table S2), which indicated that the 4nF_1_ sequences were similar to those of the other species. The organization of the *Hox* clusters in 4nF_1_ is shown in Fig. 1. The clusters can be summarized as *HoxAai*, *HoxAaii*, *HoxAaiii*, *HoxAbi*, *HoxAbii*, *HoxAbiii*, *HoxBai*, *HoxBaii*, *HoxBaiii*, *HoxBbi*, *HoxBbii*, *HoxBbiii*, *HoxCai*, *HoxCaii*, *HoxCaiii*, *HoxCbi*, *HoxCbii*, *HoxCbiii*, *HoxDai*, *HoxDaii*, and *HoxDaiii* (Table 1). Among these copies, we found that *HoxD4aiiiΨ*, *HoxD9aΨ*, and *HoxD10aΨ* in 4nF_1_ were pseudogenes (Fig. 2). Two deletions at codons 316 and 317 in the coding region of *HoxD4aiiiΨ* suggested that it was a pseudogene. The alignment of the putative *HoxD4a* sequences is shown in Fig. 2a. *HoxD9aΨ* has become a pseudogene because a stop codon prematurely terminates expression of the full-length functional product (Fig. 2b). An insertion was observed at codon 593 in the *HoxD10aΨ* coding region; alignment of the putative *HoxD10a* duplicated sequences is shown in Fig. 2c. *HoxD10aΨ* had an inserted G nucleotide compared with *HoxD10aiii*, whereas a T in *HoxD4aΨ* was replaced by a G compared with *HoxD4ai*. Thus, non-functionalization is a possible fate for some duplicated *Hox* genes. The GC levels of the pseudogenes tended to be lower than that of their counterpart genes (Additional file 1: Table S3). For instance, in 4nF_1_, the exons of the pseudogene *HoxD4aiiiΨ* exhibited a GC content of 50.1%, which was lower than that of its functional counterparts *HoxD4ai* and *HoxD4aii* (51.3%, 52.1%). As shown in Additional file 1: Table S3, the exon GC content of the pseudogene *HoxD10aiΨ* was 49.4%, which was lower than those of its putative functional counterparts *HoxD10aii* and *HoxD10aiii* (49.6% and 49.9%, respectively) in 4nF_1_. Similarly, the exon GC content of the pseudogene *HoxD9aΨ* (43.3%) was slightly lower than that of its putative functional *HoxB1b* paralogues (50.1%, 50.2%, and 50.2%). During duplication, one copy typically remains functional, whereas the other copy may lose its function, which generally leads to a decreased GC level for the non-functional gene.

### Phylogenetic relationships

**Fig. 3:**
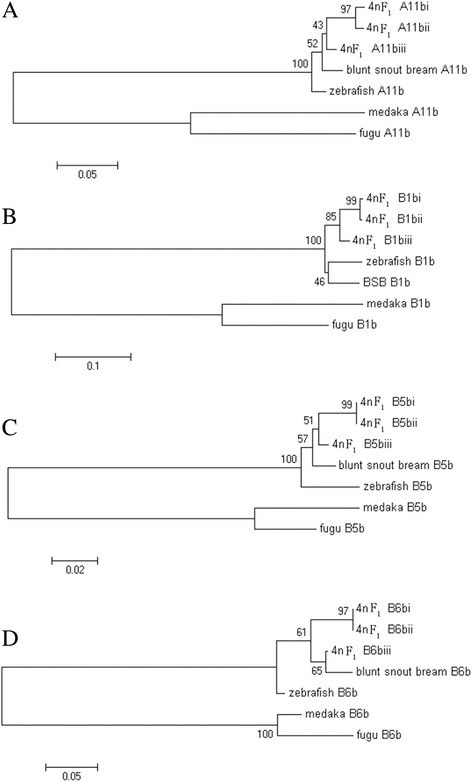
Maximum likelihood tree inferred from the alignment of amino acid sequences. This tree illustrates the phylogenetic relationships among putative *Hox* sequences in 4nF_1_ and reported orthologues from blunt snout bream (*Megalobrama amblycephala*), zebrafish (*Danio rerio*), fugu (*Fugu rubripes*), and medaka (*Oryzias latipes*). The numbers at the branch nodes indicate percentage bootstrap support for each node based on 1000 replicates. **a**–**d** *HoxA11b*, *HoxB1b*, *HoxB5b*, and *HoxB6b*

For most genes, such as *HoxA4a*, *HoxB1b*, and *HoxD10a*, three distinct orthologues of the zebrafish genes were identified in 4nF_1_. These duplicated genes shared a high identity percentage for the deduced amino acid sequences (Additional file 1: Tables S2 and S3). An identity analysis of the putative amino acid sequences suggested that the duplicated sequences were more closely related to each other than to the reported zebrafish orthologues except for the *HoxC4aiii* sequences. For instance, the percentage nucleotide identity between the *HoxA11bi*, *HoxA11bii*, and *HoxA11biii* orthologues from 4nF_1_ and *HoxA11b* from zebrafish was only 89.9%, 89.9%, and 92.4%, respectively. Conversely, the identity between the paralogues *HoxA11bi* and *HoxA11bii*, *HoxA11bi* and *HoxA11biii*, and *HoxA11bii* and *HoxA11biii* in 4nF_1_ was 98.6%, 96.4%, and 96.0%, respectively (Additional file 1: Table S2 and Fig. 2a). The identity between *HoxB1bi* and *HoxB1bii*, *HoxB1bi* and *HoxB1biii*, and *HoxB1bii* and *HoxB1biii* was 99.5%, 95.7% and 96.2%, whereas the similarity to their zebrafish orthologues was 91.0%, 90.6% and 91.5% (Additional file 1: Table S2 and Fig. 3b). These results showed that *HoxA11bi*, *HoxA11bii*, and *HoxA11biii* as well as *HoxB1bi*, *HoxB1bii* and *HoxB1biii* all share a mostly closed ancestral cluster and are true orthologues of the zebrafish genes *HoxA11b* and *HoxB1b*. Analysis of the sequences obtained for *HoxC4a* suggested that four distinct copies of this gene exist in 4nF_1_, which were named *HoxC4ai*, *HoxC4aii*, *HoxC4aiii* and *HoxC4a-1*. The putative amino acid sequence of *HoxC4a-1* shares approximately 100%, 100% and 99% similarity to those of *HoxC4ai*, *HoxC4aii*, and *HoxC4aiii*, respectively. However, the nucleotide similarity to all three sequences is 100%, which suggests the mutation was synonymous.

**Fig. 4:**
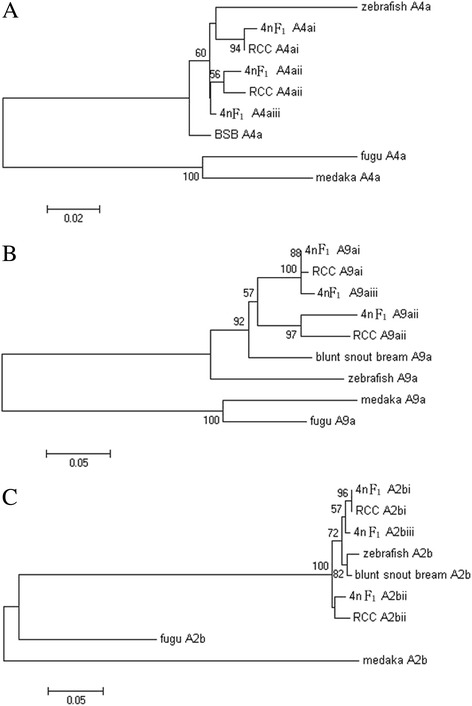
Maximum likelihood tree inferred from the alignment of amino acid sequences. This tree illustrates the phylogenetic relationships among putative *Hox* sequences in 4nF_1_, BSB, and RCC and the reported orthologues from blunt snout bream (*Megalobrama amblycephala*), zebrafish (*Danio rerio*), and medaka (*Oryzias latipes*). The number at each branch node indicates the percentage bootstrap support for that node based on 1000 replicates. **a**–**c** *HoxA4a*, *HoxA9a* and *HoxA2b.*

To evaluate the speciation of 4nF_1_, the nucleotide identity percentages among all known representatives of the *HoxA4a*, *HoxA9a*, *HoxA2b*, and *HoxD4a* gene groups in RCC, BSB, and the 4nF_1_ were examined (Table 2, Fig. 4). The identities of orthologous ‘i’ or ‘ii’ genes between 4nF_1_ and RCC were much higher than those between 4nF_1_ and BSB. For example, the nucleotide identity percentages of the orthologous *HoxA4ai*, *HoxA9ai*, *HoxA2bi*, and *HoxD4ai* genes between 4nF_1_ and RCC were 99.5%, 99.4%, 99.6% and 99.6%, respectively. Conversely, the similarity of these genes between 4nF_1_ and BSB was 97.0%, 92.3.0%, 97.2%, and 93.7%, respectively. Although similarly high identity was observed, the ‘iii’ gene in 4nF_1_ did not exhibit higher similarity to the gene in RCC or BSB for all four *Hox* sequence groups, suggesting no obvious orthologous relationship between the two species. Thus, we speculated that the ‘iii’ genes were variants of RCC or BSB genes. For example, the *HoxA4aiii*, *HoxA9aiii*, *HoxA2biii*, and *HoxD4aiii* genes from 4nF_1_ and the *HoxA4a*, *HoxA9a*, *HoxA2b*, and *HoxD4a* genes from BSB shared 98.0%, 92.2%, 97.7%, and 94.0% identity (Table 2).

**Table 2:** Percentage nucleotide identity (on the left) and percentage amino acid identity (on the right) between duplicated *Hox* coding regions in 4nF_1_, RCC, and BSB

	*HoxA4a* (%)	*HoxA9a* (%)	*HoxA2b* (%)	*HoxD4a* (%)
4nF_1_ i:4nF_1_ ii	97.4/97.5	91.8/89.5	96.9/96.9	98.3/97.7
:4nF_1_ iii	98.5/98.0	98.9/98.9	97.5/97.6	96.2/61.6
:RCC i	99.5/99.5	99.4/99.4	99.6/100.0	99.6/100.0
:RCC ii	97.2/96.6	92.3/90.1	96.5/96.6	98.3/97.7
:BSB	97.0/96.6	92.3/91.6	97.2/97.9	93.7/94.9
4nF_1_ ii:4nF_1_ iii	98.3/98.5	91.6/88.5	96.9/95.9	95.3/60.5
:RCC i	97.5/98.0	92.0/89.0	96.8/96.9	98.3/97.7
:RCC ii	98.8/98.5	94.9/92.1	98.3/97.6	100.0/100.0
:BSB	96.7/97.0	91.0/86.4	96.4/96.3	93.3/92.7
4nF_1_ iii:RCC i	98.7/98.5	99.1/98.4	97.4/97.6	95.9/61.6
:RCC ii	98.2/98.5	92.2/89.0	96.5/95.9	95.3/60.5
:BSB	98.0/98.5	92.2/90.6	97.7/97.3	94.0/59.4
RCC i:RCCii	97.4/97.0	92.5/89.5	96.6/96.6	98.3/97.7
:BSB	97.2/97.0	92.5/91.1	97.1/97.9	94.0/94.9
RCC ii:BSB	96.9/97.0	91.5/88.0	96.2/96.6	93.3/92.7

Values before slashes (/) denote nucleotide identity, values after slashes denote amino acid identity

## Discussion

### The structure of cloned *Hox* gene sequences

Prior PCR surveys and genomic library screening have identified interesting variability in *Hox* gene content among teleosts [12, 15, 16, 20, 21]. Luo et al. [22] estimated there were 14–16 *Hox* gene clusters in goldfish. Our data suggested 18–21 *Hox* gene clusters were present in 4nF_1_, with each was located on a different acrocentric chromosome. The *Hox* gene clusters in 4nF_1_ were approximately the sum of the clusters in RCC and BSB, except that some clusters were lost. The topology of the *Hox* gene maximum likelihood tree (Fig. 3) further suggested that some of the *Hox* genes orthologous to zebrafish genes were present in two copies in RCC, one copy in BSB, and three copies in 4nF_1_. However, the third copy did not exhibit notably higher similarity to the gene in RCC or BSB. We speculated that variation and reorganization of the genome likely occurred during polyploidization, resulting in new copies in 4nF_1_. This might be evidence that allopolyploidization induces a variety of rapid genomic changes in a 4nF_1_ population [23, 24]. Using sequence alignment in 4nF_1_, we isolated 32 fragments that can be characterized as *HoxA, HoxB*, *HoxC*, and *HoxD* family genes. However, amplified RCC and BSB DNA were only characterized as the *HoxA* and *HoxD* genes. We speculated that the increase in the number of 4nF_1_ genes might be related to polyploidization. This situation was also observed in our previous study [25, 26]; the number of 4nF_1_ fragments increased, and some genes from RCC and BSB were lost. At present, although we have no precise data explaining this outcome, we speculate that allotetraploidization might lead to rapid changes in 4nF_1_ genome diversity. Our study is the first to evaluate the organization of *Hox* clusters in a 4nF_1_ population. This theory is also strongly supported by other studies examining *Hox* genes [22], other gene families [27], and DNA content [28].

### The significance of polyploidization

Polyploidization likely increases genomic variation rates, which can result in the formation of new polyploid species [29]. First, the process of polyploidization can itself generate species that are reproductively isolated from their diploid progenitors, increasing the number of species as a by product. For example, several studies have indicated that a polyploidization event occurred in an ancestor of teleost fish shortly after this lineage diverged from the lineage leading to tetrapods [30–32]. Second, an entirely different trait can result in increased rates of polyploidization [6]. Synonymous mutations increase genomic variation. For example, the putative amino acid sequence of *HoxC4a-1* shares approximately 100%, 100%, and 99% similarity with those of *HoxC4ai*, *HoxC4aii*, and *HoxC4aiii*, respectively. The identity of their nucleotide sequences is 100%. In the polyploidization process, genome duplication produces abundant genomic DNA, so the organism maintains the dosage balance or rapidly stabilizes the duplicated genomes via retention/exclusion of redundancy. Lynch et al. [33] suggested there are three outcomes in the evolution of duplicate genes: non-functionalization, neo-functionalization and sub-functionalization. Interestingly, we found some pseudogenes in 4nF_1,_ such as *HoxD4aiiiΨ*, *HoxD9aΨ* and *HoxD10aΨ*. Pseudogenes are formed either by random mutations that create stop codons and prematurely terminate full-length functional product expression or by insertions/deletions that shift the reading frame, rendering the translated protein non-functional. We speculate that dosage effects generated selection pressure from the loss of *Hox* genes or the formation of pseudogenes after whole genome duplication. This pressure is consistent with the expectation that there are *Hox* clusters in the 4nF_1_ genome that have lost functional *Hox* genes due to the reduction of redundancy following the polyploidization event. However, 4nF_1_ required genetic recombination, mutation, and pseudogenization to reduce the amount of incompatible genetic material and improve fertility [34]. Thus, we unexpectedly obtained autotetraploids with greater fertility among the 4nF_1_ progeny, and we successfully established an autotetraploid fish line [35]. Our characterization of the *Hox* gene clusters in tetraploid hybrids improves our understanding of the evolutionary processes occurring after *Hox* gene duplication in vertebrates.

## Conclusions

We identified three copies of *Hox* genes in 4nF_1_, two copies in RCC and one copy in BSB. In addition, obvious variation and pseudogene generation were observed in some 4nF_1_ *Hox* genes. These results reveal the effects of polyploidization on the organization and evolution of *Hox* gene clusters in fish and also help to clarify aspects of vertebrate genome evolution.

## Methods

### DNA extraction

Specimens of 4nF_1_ (4n = 148), RCC (2n = 100), and BSB (2n = 48) were obtained from the Engineering Research Center of Polyploid Fish Breeding and Reproduction of the State Education Ministry at Hunan Normal University. Fish treatments were carried out according to the regulations for protected wildlife and the Administration of Affairs Concerning Animal Experimentation, and approved by the Science and Technology Bureau of China. Approval from the Department of Wildlife Administration was not required for the experiments conducted in this paper. The fish were deeply anesthetized with 100 mg/L MS-222 (Sigma-Aldrich, St Louis, MO, USA) before dissection. Narcotic drugs was fed before blood sampling. Total genomic DNA was isolated from peripheral blood cells using the standard phenol chloroform extraction procedures described by Sambrook et al. [36].

### Cloning and sequencing of *Hox* genes

We amplified fragments of *Hox* genes from genomic DNA by PCR amplification using several combinations of degenerate primers (Table 1). PCR was performed in 50-μL reaction volumes using Taq DNA polymerase (TaKaRa, Dalian, China). A typical PCR programme consisted of a denaturation step at 94°C for 5 min; 35 cycles of 98°C for 15 s, 55°C for 45 s and 72°C for 1 min; and a final elongation step at 72°C for 10 min. PCR products were cloned into a T vector and sequenced with an automated ABI 3700 DNA sequencer. The sequences were BLAST searched against the non-redundant protein database maintained at the National Center for Biotechnology Information (www.ncbi.nlm.nih.gov) to determine their identity.

### Sequence comparison and analysis

Sequence homology and variation among the fragments amplified from RCC, BSB and the 4nF_1_ were analysed in BioEdit [37, 38]. Partial DNA sequences for each gene were verified using a BLASTx search. To increase the probability of detecting duplicated paralogues and circumventing PCR errors, we sequenced 20 clones for each gene from 4nF_1_, RCC and BSB. The obtained sequences were screened for *Hox* gene fragments using different BLAST searches (BLASTn, BLASTp, and BLASTx) against GenBank (http://www.ncbi.nlm.gov/Blast.cgi). Then, we evaluated the organization of the 4nF_1_ *Hox* clusters compared to RCC and BSB to characterize the *Hox* genes.

### Phylogenetic analysis

Using Clustal X 1.81, the derived amino acid sequences of these fragments were aligned with the *Hox* genes from BSB, zebrafish, fugu, medaka and other teleosts retrieved from GenBank [38]. Regions of sequences that were difficult to align were removed from the alignment. Gaps were also removed from the alignment. The maximum likelihood method implemented in the online software RAxML was used to construct a phylogenetic tree [39].

## Additional file

Additional file 1: Table S1. The PCR Gene-specific degenerate primers. Gene-specific degenerate primers designed based on the alignment and identification of consensus orthologous *Hox* gene sequences from zebrafish (*Danio rerio*), medaka (*Oryzias latipes*), pufferfish (*Fugu rubripes*), mouse (*Mus musculus*), cichlids, and humans (*Homo sapiens*). **Table S2.** The Percentage of the amino acid. Percentage amino acid identity between paralogous *Hox* sequences obtained from 4nF_1_ and reported orthologues from zebrafish, fugu, and medaka. **Table S3.** Comparison of GC levels among duplicated genes. (DOCX 20 kb)

## Abbreviations

4nF_1_: Allotetraploid F_1_ hybrids
BSB: Blunt snout bream
RCC: Red crucian carp
