# Supplementary material for: Hox genes reveal genomic DNA variation in tetraploid hybrids derived from Carassius auratus red var. (female) × Megalobrama amblycephala (male)
Source: BMC Genet. 2017 Oct 11;18:86. doi: 10.1186/s12863-017-0550-2 (PMC5637053; doi:10.1186/s12863-017-0550-2)
Supplement: Additional file 1: Table S1. — The PCR Gene-specific degenerate primers. Gene-specific degenerate primers designed based on the alignment and identification of consensus orthologous Hox gene sequences from zebrafish (Danio rerio), medaka (Oryzias latipes), pufferfish (Fugu rubripes), mouse (Mus musculus), cichlids, and humans (Homo sapiens). Table S2. The Percentage of the amino acid. Percentage amino acid identity between paralogous Hox sequences obtained from 4nF1 and reported orthologues from zebrafish, fugu, and medaka. Table S3. Comparison of GC levels among duplicated genes. (DOCX 20 kb) [file 12863_2017_550_MOESM1_ESM.docx]

**Additional file 1**

**Table S1** The PCR Gene-specific degenerate primers. These primers designed based on the alignment and identification of consensus sequences of the orthologous *Hox* genes from zebrafish (*Danio rerio*), medaka (*Oryzias latipes*), pufferfish (*Fugu rubripes*), mouse (*Mus musculus*), Cichlid, and humans (*Homo sapiens*).

| **No** | **Gene** | **Forward primer** | **Reverse primer** |
| --- | --- | --- | --- |
| 1 | *HoxA4a* | TACATTTGACAGAGGAGTAGGT | TGTGSTCYTTCTTCCACTTCA |
| 2 | *HoxA9a* | TCGGCCCCTGCACATTC | CATCCTGCGGTTTTGGAACCANAT |
| 3 | *HoxA2b* | CTCGCTGAGTGCCTGACAT | GAGAAAACRTTGAAGTCCTGYAAA |
| 4 | *HoxA11b* | GGATGATGGATTTTGATGAGC | TTTTCYTTGTTGATGTARACACTGA |
| 5 | *HoxB1b* | ATAGTTACATTCCCGAGG | ACCTGCGTTTCGTTCAGTTC |
| 6 | *HoxB5b* | ACTCSTTCTCRGGGCGYTA | TTTCAGTTTATTGTCCTTCTTCCA |
| 7 | *HoxB6b* | AACCGCTGACCGCTCGTGC | GTCAAACACAGAGCGTGCGATAT |
| 8 | *HoxC4a* | GGACTCTAACTACATCGATC | GAGGACGAGGATCTGACTTTG |
| 9 | *HoxD4a* | AGAGCCCAGGCTACTACA | CTTTCGTGTTTGGTAGTTTGTG |
| 10 | *HoxD9a* | CGAGGCTGAGGATGTGTACG | CAGATTTTCACCTGGCGCTC |
| 11 | *HoxD10a* | GGA ATGCAAACCTGTGGACT | TCKGCTCATCTTCTTGAGTTTCA |

**注：R=A/G; Y=C/T; S=G/C; W=A/T; K=G/T; M=A/C; B=C/G/T; D=A/G/T; H=A/C/TandV=A/C/G.**

**Table S2** The Percentage of the amino acid. The Percentage amino acid identity between the paralogous *Hox* sequences obtained from 4nF_1_ and the reported orthologs from zebrafish, fugu, and medaka.

| **Gene** | **Duplicated sequences** | **Blunt snout bream** | **Zebrafish** | **Fugu** | **Medaka** |
| --- | --- | --- | --- | --- | --- |
| *HoxA11bi* | vs.ii:98.6 | 93.1 | 89.9 | 53.8 | 54.5 |
| *HoxA11bii* | vs.iii:96.0 | 93.1 | 89.9 | 54.2 | 54.9 |
| *HoxA11biii* | vs.i:96.4 | 95.6 | 92.4 | 55.5 | 54.0 |
| *HoxB1bi* | vs.ii:99.5 | 89.2 | 91.0 | 37.8 | 34.3 |
| *HoxB1bii* | vs.iii:96.2 | 89.7 | 90.6 | 37.2 | 34.3 |
| *HoxB1biii* | vs. i: 95.7 | 90.6 | 91.5 | 36.8 | 34.1 |
| *HoxB5bi* | vs.ii:100.0 | 96.0 | 94.1 | 69.4 | 66.7 |
| *HoxB5bii* | vs.iii:96.4 | 96.0 | 94.1 | 69.4 | 66.7 |
| *HoxB5biii* | vs.i:96.4 | 98.0 | 96.0 | 69.7 | 67.1 |
| *HoxB6bi* | vs.ii: 100.0 | 90.9 | 92.1 | 62.2 | 63.8 |
| *HoxB6bii* | vs.iii:96.0 | 90.9 | 92.1 | 62.2 | 63.8 |
| *HoxB6biii* | vs.i:96.0 | 96.1 | 96.0 | 61.3 | 61.9 |
| *HoxC4ai* | vs.ii:100.0 | 100.0 | 99.5 | 90.7 | 87.3 |
| *HoxC4aii* | vs.iii:99.0 | 100.0 | 99.5 | 90.7 | 87.3 |
| *HoxC4aiii* | vs.i:99.0 | 99.0 | 98.6 | 89.8 | 86.4 |
| *HoxC4a-1* | 100/100/99.0 | 100.0 | 99.5 | 90.7 | 87.3 |
| *HoxD9a*Ψ | - | 70.9 | 71.2 | 39.2 | 38.9 |
| *HoxD10ai*Ψ | vs.ii:96.7 | 96.4 | 69.3 | 43.9 | 47.5 |
| *HoxD10aii* | vs.iii: 96.7 | 97.3 | 95.6 | 67.8 | 71.2 |
| *HoxD10aiii* | vs.i: 72.6 | 96.4 | 93.7 | 67.1 | 69.5 |

Ψ denotes pseudogene.

**Table S3** Comparison of the GC level in twin genes pairs.

| **Species** | **Gene i** | **GC%** | **Gene ii** | **GC%** | **Gene iii** | **GC%** |
| --- | --- | --- | --- | --- | --- | --- |
| **4nF_1_** | *HoxB1bi* | 50.1 | *HoxB1bii* | 50.2 | *HoxB1biii* | 50.2 |
|  | *HoxD4ai* | 51.3 | *HoxD4aii* | 52.1 | *HoxD4aiii*Ψ | 50.1 |
|  | *HoxD9a*Ψ | 43.3 | **-** | - | **-** | - |
|  | *HoxD10ai*Ψ | 49.4 | *HoxD10aii* | 49.6 | *HoxD10aiii* | 49.9 |

Ψ denotes pseudogene.
